# Ultrasensitive and Highly Stretchable Multiple-Crosslinked Ionic Hydrogel Sensors with Long-Term Stability

**DOI:** 10.1007/s40820-023-01015-7

**Published:** 2023-02-15

**Authors:** Jin-Young Yu, Seung Eon Moon, Jeong Hun Kim, Seong Min Kang

**Affiliations:** 1 Department of Mechanical Engineering, Chungnam National University, Daejeon, 34134 Korea; 2 Emerging Nano-Materials Research Section, Electronics and Telecommunications Research Institute, Daejeon, 305-700 Republic of Korea

**Keywords:** Hydrogel sensors, Biocompatibility, Multifunction, High-sensitivity sensors, Multiple-crosslink association

## Abstract

Flexible hydrogels are receiving significant attention for their application in wearable sensors. However, most hydrogel materials exhibit weak and one-time adhesion, low sensitivity, ice crystallization, water evaporation, and poor self-recovery, thereby limiting their application as sensors. These issues are only partly addressed in previous studies. Herein, a multiple-crosslinked poly(2-(methacryloyloxy)ethyl)dimethyl-(3-sulfopropyl)ammonium hydroxide-co-acrylamide) (P(SBMA-co-AAm)) multifunctional hydrogel is prepared via a one-pot synthesis method to overcome the aforementioned limitations. Specifically, ions, glycerol, and 2-(methacryloyloxy)ethyl)dimethyl-(3-sulfopropyl)ammonium hydroxide are incorporated to reduce the freezing point and improve the moisture retention ability. The proposed hydrogel is superior to existing hydrogels because it exhibits good stretchability (a strain of 2900%), self-healing properties, and transparency through effective energy dissipation in its dynamic crosslinked network. Further, 2-(methacryloyloxy)ethyl)dimethyl-(3-sulfopropyl)ammonium hydroxide as a zwitterion monomer results in an excellent gauge factor of 43.4 at strains of 1300–1600% by improving the ion transportability and achieving a strong adhesion of 20.9 kPa owing to the dipole–dipole moment. The proposed hydrogel is promising for next-generation biomedical applications, such as soft robots, and health monitoring.

**Supplementary Information:**

The online version contains supplementary material available at 10.1007/s40820-023-01015-7.

## Highlights

- A multiple-crosslinked poly(2-(methacryloyloxy)ethyl)dimethyl-(3-sulfopropyl)ammonium hydroxide-co-acrylamide multifunctional hydrogel was prepared via a one-pot synthesis method.
- The proposed hydrogel exhibits water retention, antifreeze properties, self-healing, and transparency as well as improved strength, good adhesiveness, and a high gauge factor.

**Supplementary Information:**

The online version contains supplementary material available at 10.1007/s40820-023-01015-7.

## Graphical abstract

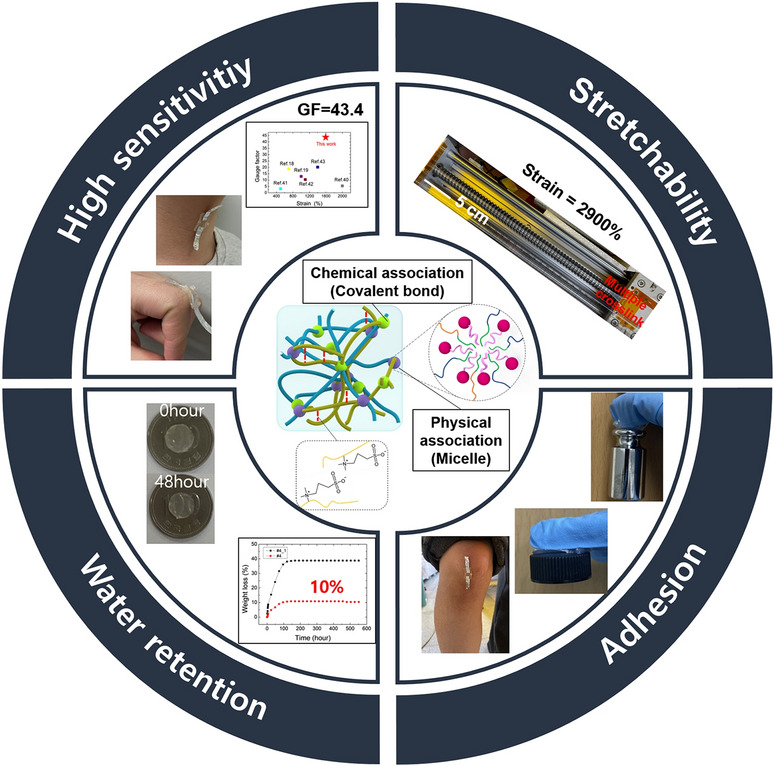

## Introduction

Hydrogels have been extensively employed in biomedical applications, such as wearable electronics [1, 2], tissue engineering [3, 4], soft robotics [5, 6], and drug delivery [7]. In particular, hydrogels are attracting increasing attention as wearable materials because they are soft, flexible, stretchable, transparent, and biocompatible [8, 9]. However, their applications are limited by several drawbacks, such as poor strength, weak [10] and one-time adhesion, poor self-recovery, water evaporation, ice crystallization, and low sensitivity. These issues have only been partially resolved in previous studies and must be overcome in the future applications of hydrogels.

Recently, novel hydrogel designs based on various materials, such as carbon nanotubes (CNTs), graphene, and nanocomposites, have been proposed to overcome the aforementioned limitations. CNTs improve the conductivity and mechanical properties of hydrogels; however, their inherent hydrophobicity complicates the manufacturing processes, and they result in low stretchability owing to their nonuniform entanglement and opacity [11–14]. Although graphene has an exceptionally high electrical conductivity, sophisticated redox processes disrupt its structure; microscale patterning on a substrate is challenging and hinders production; and the surface of graphene is black, similar to that of CNTs [15, 16]. Nanocomposites possess biocompatibility, excellent deformation ability, and high strength; however, their electrical conductivity and sensitivity are low [15, 17, 18].

Previously, Wang et al. reported AFPs-PAM/PVA hydrogels with a high gauge factor (GF), self-adhesion, anti-icing properties, and strong modules [19]. Furthermore, Nie et al. reported PCP nanocomposite hydrogels, which had antifreeze properties, sensitivity to strain, and excellent mechanical properties [20]. However, transparency, self-recovery, and moisture retention were not realized in either study.

This study investigated the one-pot synthesis of high-performance multiple-crosslinked poly(2-(methacryloyloxy)ethyl)dimethyl-(3-sulfopropyl)ammonium hydroxide (SBMA)-co-acrylamide (AAm)) (P(SBMA-co-AAm)) hydrogels. Ionically conductive hydrogels with a high water content lose flexibility and conductivity under evaporation and freezing conditions, thereby limiting their application as flexible sensors. To overcome these limitations, we developed a cosolvent consisting of water and glycerol to improve the water retention and antifreeze properties of the hydrogel, together with the addition of NaCl ions and SBMA. The hydrogel was polymerized to form multiple crosslinks with covalent and hydrophobic bonds. Subsequently, the hydrogel was characterized to determine its chemical structure and mechanical properties. In particular, the reversible bonding property of the hydrophobic micelle formed using lauryl acrylate (LA) and sodium dodecylbenzenesulfonate (SDBS) enhanced the mechanical properties of the gel through energy dissipation [21]. Its transparency, adhesion to different substrates, water retention, and antifreeze properties were also measured. Furthermore, the electrical properties of the hydrogel were tested, and it was employed for various sensing applications.

A zwitterionic polymer includes both negative and positive groups in its chains. Representative zwitterion polymers, such as 2-(methacryloyloxy)ethyl)dimethyl-(3-sulfopropyl)ammonium hydroxide (SBMA), have sulfonic acid groups as the negative groups and ammonium cation groups as the positive groups; these act as dipolarized sites and enhance ion transportation [22, 23]. Furthermore, zwitterionic polymers have been confirmed to exhibit a self-adhesive ability, which is an important requirement in wearable devices, owing to the dipole–dipole moment between the hydrogel and the substrate.

## Materials and Methods

### Materials

Deionized (DI) water, glycerol (≥ 99%), SDBS, and sodium chloride (NaCl, powder, ≥ 99.5%) were purchased from DAEJUNG Chemical. LA (liquid, 90%), AAm (powder, 99%), SBMA (powder, 95%), N,N′-methylenebis (acrylamide) (BIS, powder, 99%), and 2-hydroxy-2-methylpropiophenone (photoinitiator, liquid, 97%)(PI) were purchased from Sigma-Aldrich.

### Fabrication of Multiple-Crosslinked P(SBMA-co-AAm) Hydrogel

First, 1.5 g of DI Water, 1.5 g of glycerol, 0.08 g (m: 0.076) of SDBS, and 0.02 g (m: 0.027) of LA were mixed in a glass bottle using a magnetic stirrer at 320 rpm for at least 30 min. Subsequently, AAm and SBMA were added according to the recipe (Fig. S1). The solution was further stirred for 1 h until it was mixed well and became transparent. Next, 0.0005 g (m: 0.001) of BIS was added to the solution and mixed for at least 15 min using the magnetic stirrer. Thereafter, 0.35 g (m: 1.996) of NaCl was added to the solution and dissolved. Subsequently, 0.01 g (m: 0.020) of PI was dropped in the solution and mixed for at least 30 min. Finally, the solution was poured into a 20 mm (length) × 10 mm (width) × 1 mm (height) mold, placed in an ultraviolet (UV) curing machine, and cured for 2 h.

### Characterization

#### UV–Visible (Vis) Absorption Spectroscopy

The transmittance was measured using a UV − vis spectrophotometer (Lambda 365, PerkinElmer) in the wavelength range of 300–800 nm.

#### Mechanical Tests

The mechanical properties of the P(SBMA-co-AAm) zwitterionic hydrogels were measured using an electronic universal tensile testing machine (MCT-1150, AND). The tensile and uniaxial speeds were set at 100 mm min^−1^. The sample had a dog-bone shape with a cross-section of 1 mm (width) × 1.2 mm (thickness). Four cycle tests were performed with 600% strain. The elastic modulus was measured with an additional 4 mm of tension from the initial length.

#### Adhesion Shear Tests

A lap shear test was performed using an electronic universal tensile testing machine (MCT-1150, AND) as in the mechanical test. Samples with dimensions of 20 mm (length) × 10 mm (width) × 1 mm (thickness) were prepared. To measure the lap shear force, a hydrogel was sandwiched between two substrates of the same type. The lap shear rate was maintained at 100 mm min^−1^. The adhesive strength was calculated based on the maximum load on the area of the superimposed hydrogel. Repeated tests were conducted with a period of 5 min between cycles.

#### Electrical Measurements

The electrical properties of the hydrogel were measured using an LCR Meter (U1733, Keysight) via alternative current (AC) impedance spectroscopy (electrochemical impedance spectroscopy, EIS). The test frequency was 1 kHz. The hydrogel had dimensions of 20 mm (length) × 10 mm (width) × 1 mm (thickness), and the electrode had dimensions of 25 mm (length) × 5 mm (width) × 0.05 mm (thickness). The electrode was composed of aluminum foil. The resistance values were recorded using a data logger for the Agilent Handheld LCR Meter (U1730C). The sensitivity (*α*) and GF values were calculated using the obtained resistance values as follows:

1 $$\alpha = \frac{R-{R}_{0}}{{R}_{0}} \times 100$$

where *R* is the resistance value according to the length, and *R*_0_ is the initial resistance value.

2 $$\varepsilon = \frac{\Delta L}{{L}_{0}}\times 100$$

where ε is the strain, $\Delta L$ is the increased length, and ${L}_{0}$ is the initial length.

3 $$\mathrm{GF}=\frac{\Delta \mathrm{\alpha }}{\Delta\upvarepsilon }$$

where $\Delta \mathrm{\alpha }$ and $\Delta\upvarepsilon$ are specified value of the applied interval.

Another LCR meter (E4980AL Precision LCR Meter, Keysight) was used to measure the sensor response rate. Furthermore, conductivity was measured using electrochemical impedance spectroscopy (EIS; ZIVE SP2, Wonatech). The frequency of the test was 100 kHz–1 Hz. The disturbance voltage was 10 mV. The ionic conductivity was calculated using the following equation:

4 $$\upsigma = \frac{t}{R \times A }$$

where *t* is the thickness of the hydrogel, *R* is the resistance of the hydrogel, and *A* is the contact area between the hydrogel and electrode.

#### Measurement of Water Retention Properties

Samples without and with glycerol were prepared using the method described above, and their water retention properties were compared. Both samples had dimensions of 90 mm (diameter) × 1 mm (thickness). Further, both samples were stored at 25 °C, and each sample was weighed daily. The weight loss (%) was calculated as follows [24]:

5 $$\mathrm{Weight Loss}\left(\text{\%}\right)=-\frac{\mathrm{Mass}\left(\mathrm{hydrogel},\mathrm{ time}=e\right)-\mathrm{Mass}\left(\mathrm{hydrogel},\mathrm{ time}=0\right)}{\mathrm{Mass}\left(\mathrm{hydrogel},\mathrm{ time}=0\right)}\times 100$$

#### Antifreeze Test

The hydrogel samples were placed in a freezer at − 17 °C, and the sensitivity and flexibility were compared with those of the original sample stored at 25 °C. The freezing point was determined through differential scanning calorimetry (DSC 1, Mettler-Toledo). The test was started at 20 °C and progressed to − 50 °C at a rate of 10 °C min^−1^.

#### Fourier-Transform Infrared (FT-IR) Spectroscopy Measurements

FT-IR spectroscopy measurements were performed using IFS66V/S, HYPERION 3000 system, and ALPHA equipment manufactured by Bruker Optiks (Germany). The chemical structures were evaluated, and the chemical stability was measured by preparing two types of test samples: one-month-old and less-than-24-h-old hydrogels.

## Results and Discussion

### Preparation of the Multiple-Crosslinked P(SBMA-co-AAm) Hydrogel

**Fig. 1:**
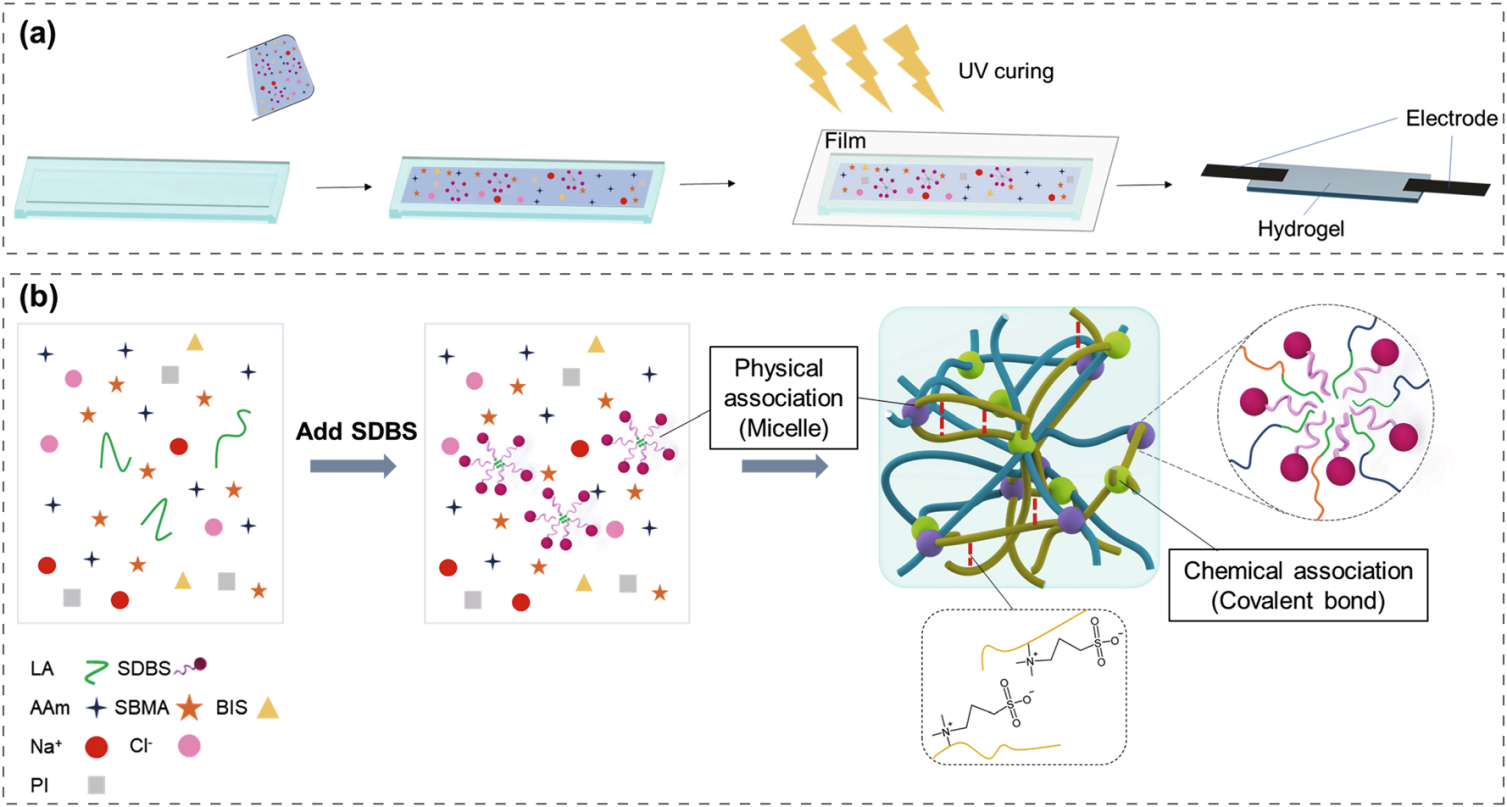
**a** Hydrogel fabrication process. **b** Schematic of the preparation process of multiple-crosslinked P(SBMA-co-AAm) hydrogels

A water and glycerol mixture were used to minimize the evaporation of water. SDBS and LA were added to establish a hydrophobic association with a reversible dynamic bond, and NaCl was added to achieve a high ionic conductivity as well as a low freezing point. Thereafter, the SBMA, AAm monomer, and BIS crosslinker were diluted and copolymerized via free-radical polymerization under UV irradiation (Fig. 1a).

**Fig. 2:**
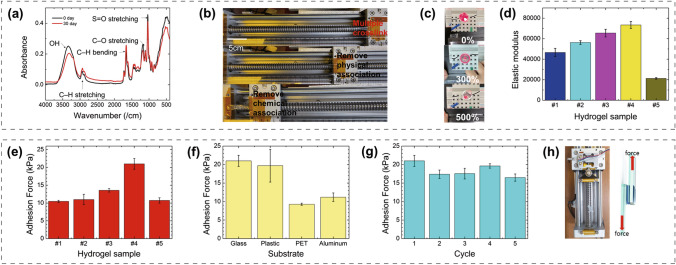
**a** FT-IR spectra of the multiple-crosslinked P(SBMA-co-AAm) hydrogels. **b** Stretchability differences according to the hydrophobic or chemical association. **c** Comparison of the intensity of light according to the strain. **d** Elastic modulus depending on the hydrogel composition. **e** Adhesion force on glass at different compositions. **f** Adhesion force of #4 hydrogel to various substrates. **g** Adhesion force of #4 hydrogel on glass at various cycles. **h** Adhesive experimental design

The multiple-crosslinked P(SBMA-co-AAm) hydrogel consists of chemical bonds with an organic crosslinker and hydrophobic associations with the long alkyl chains of LA. The hydrogel exhibits self-recovery, strong adhesion, high sensitivity, antifreeze properties, water retention, and high transmittance. The hydrophobic segments of LA form into micelle-like aggregates that act as dynamic crosslinking points in the hydrogel via molecular entanglement (Fig. 1b) [21, 25]. This hydrophobic association affords stretchability and a self-healing ability, thereby enabling the effective dissipation of energy and restoration of the mechanical properties under an external force because of the inherent mobility and reversibility [26]. Consequently, all components in the cosolvent system can be cured through the one-pot synthesis method. SBMA, as a zwitterion, is biocompatible because it is derived from maltose and glycine betaine sourced from plants [27]. The intermolecular negative and positive groups in the zwitterionic SBMA react with each other to form an electrical noncovalent bond [28]. The zwitterion active sites of the charged groups accelerate ion migration and thereby increase the conductivity. However, if the SBMA is not appropriately dispersed, polymerization cannot be achieved owing to the internal bonding among the charged groups. In the experiment, the hydrogel composed of only SBMA exhibited gum-like characteristics and could not maintain its shape. Therefore, the SBMA must be uniformly dispersed by adjusting its proportion with AAm. Samples were named as #1–#5 according to the ratio between SBMA and AAm (Fig. S1). Their proportions were adjusted to realize the maximum strength and adhesion (Fig. 2d, e). Figure S1 presents the hydrogel composition, including one control group without SBMA and other groups with sequentially increasing amounts of SBMA. The successful synergistic combination of multiple crosslinks, a cosolvent system, and zwitterions afforded significant advantages.

### Properties of the Multiple-Crosslinked P(SBMA-co-AAm) Hydrogel

#### Formation Mechanism

To confirm the chemical structure of the hydrogels, FT-IR spectra of as-prepared and one-month-old samples are presented (Fig. 2a). The two curves are almost identical, indicating that the multiple-crosslinked P(SBMA-co-AAm) hydrogel is chemically stable. The peak at 1036 cm^−1^ is attributed to S=O stretching. The highest-intensity peak at 1143 cm^−1^ is attributed to C–O stretching. The intense peak between 1400 and 1454 cm^−1^ is attributed to C–H bending. Further, the low-intensity, narrow curve between 2800 and 3000 cm^−1^ suggests that C–H stretching is present in the bifurcated curves. The broad peak at 3300 cm^−1^ is attributed to OH.

#### Mechanical Performance and Adhesion

Hydrogels that are crosslinked only chemically have low strength and poor mechanical and deformation properties because they lack an energy dissipation mechanism [21]. This study introduced multiple crosslinks in the hydrogel with chemical associations to form a covalent bond as well as physical associations to realize energy dissipation. Figure S2 shows the stable stress–strain curve of multiple-crosslinked P(SBMA-co-AAm). Excluding the first cycle owing to the influence of hysteresis, curves of the same shape were obtained from the second to the fourth cycle, which confirmed that the hydrogel had uniform and stable physical properties. Multiple-crosslinked P(SBMA-co-AAm) showed a total strain of 2900%, which is higher than those of the hydrogels with only chemical (strain = 2200%) associations or hydrophobic associations (strain = 200%) (Fig. 2b). The luminance intensity of the light-emitting diode (LED) varied with the elongation of the hydrogel because narrow ion-transportation paths resulted in increased resistance (Fig. 2c). This is the basic principle of hydrogel strain sensors. When a hydrogel containing ions is lengthened, the ion channels become narrow and the resistance increases. This change in resistance can be measured with respect to the displacement (Fig. S3).

The total number of molality of SBMA and AAm was fixed, and their proportions were adjusted to obtain the maximum strength and adhesion (Fig. 2d, e). Initially, the Young’s modulus and adhesion strength improved in proportion to the increasing SBMA content, following which they decreased rapidly. The sample that exhibited such a decrease is hydrogel #5.

A low Young’s modulus was obtained with a nonuniform monomer distribution, and the adhesion rapidly decreased because dipole–dipole interactions with the substrate could not be achieved owing to the agglomeration of charged groups. In hydrogel #4, most of the SBMA and AAm participated in the chemical and physical associations because both were well dispersed in the polymer network owing to the appropriate distribution and suitable proportions of the components, resulting in the highest strength and adhesion force. The hydrogel composed of only SBMA without AAm did not retain its shape as in another report [29]. Guo et al. studied the elastic modulus of hydrogels with respect to the SBMA ratio. In their study, which is similar to ours, the elastic modulus increased and then decreased as the SBMA ratio increased [28]. The results confirmed that hydrogel #4 is suitable for applications and that the optimization of AAm and SBMA is important.

The optimal composition (i.e., hydrogel #4) exhibited a Young’s modulus of 73.4 kPa and an adhesion of 20.99 kPa with the glass substrate. These results suggest that the hydrogel has various potential applications owing to its ability to adhere to different substrates (Fig. 2f). Further, the hydrogel could be attached to a glass substrate and detached from it repeatedly owing to its reversible physical interactions (Fig. 2g, h), and its adhesion strength was maintained during cycle tests.

#### Water Retention

**Fig. 3:**
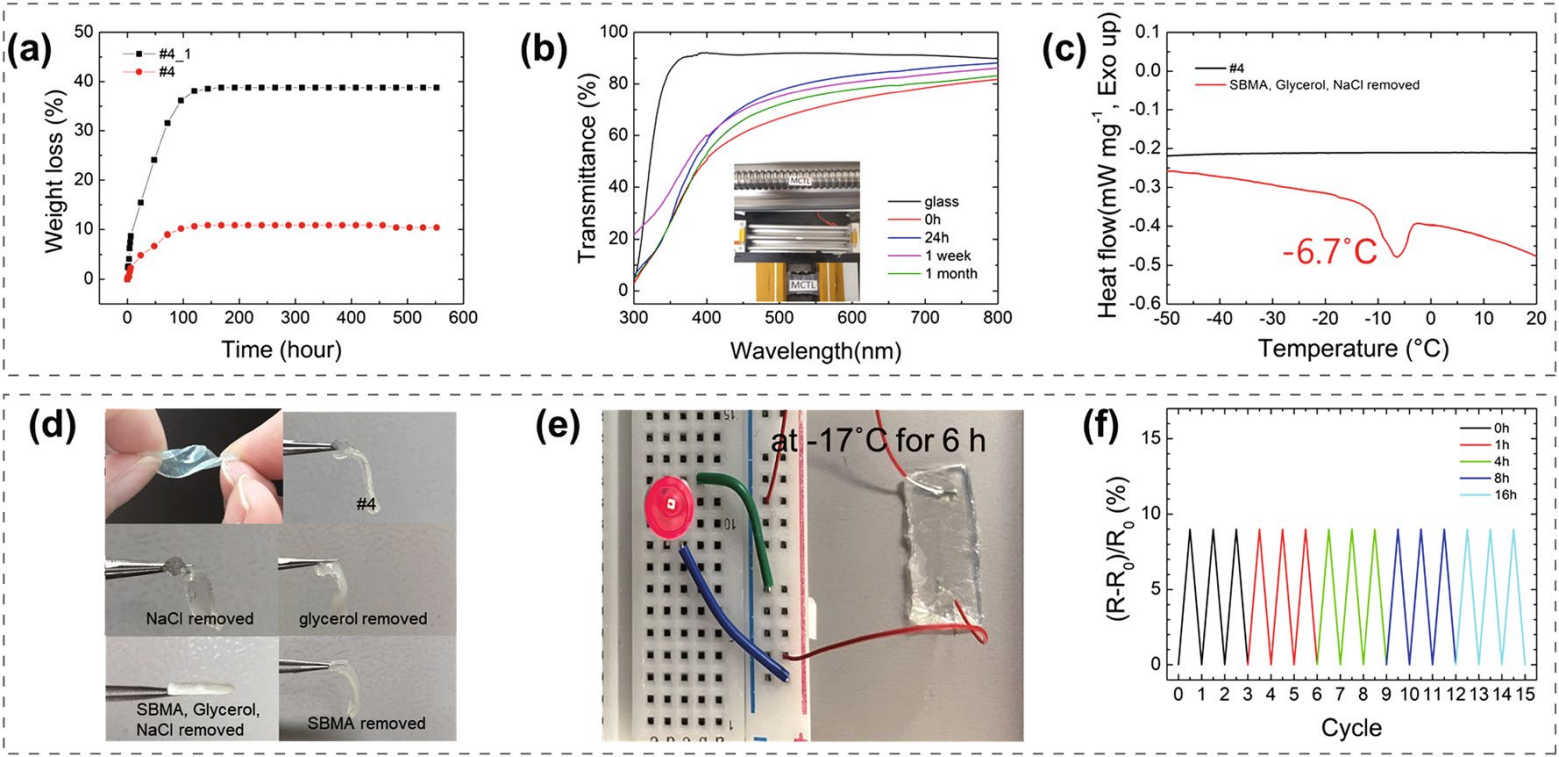
Performance of #4 hydrogel: **a** Weight loss (%) of hydrogel with and without glycerol over 552 h. **b** Wavelength transparency from 300 to 800 nm. **c** DSC graphs of #4 hydrogel and #4 hydrogel without SBMA, glycerol, and NaCl. **d** Photographs demonstrating freezing point change at the low temperature (− 17 °C) depending on the contents of NaCl, glycerol, and SBMA. **e** LED luminescence intensity testing through circuit configuration at − 17 °C. **f** Sensitivity at the low temperature (− 17 °C) at a strain of 10%

Typically, water evaporation in a hydrogel negatively impacts the durability of the hydrogel, and various studies have been conducted to enhance the water retention performance of hydrogels [24, 30]. In this study, glycerol, which is biocompatible and hygroscopic, was added to increase water retention, and its effects were elucidated. The #4 hydrogel containing glycerol showed a 10.39% weight loss over 552 h, whereas the #4_1 hydrogel without glycerol showed a 38.79% weight loss (Fig. 3a). The dimension of #4 hydrogel remained approximately the same from 0 to 48 h (Fig. S4), whereas that of the hydrogel without glycerol showed a significant difference. This result indicates that the hydrogel containing glycerol became significantly more resistant to water vaporization than that without glycerol.

#### Transparency

Wearable devices must be transparent to enable the observation of a sensing site [31]. We characterized the optical transparency at wavelengths of 300–800 nm for the multiple-crosslinked P(SBMA-co-AAm) (Fig. 3b). The hydrogel showed excellent performance and durability even after one month. The maximum optical transparency of the hydrogel was 80.6% at 400–800 nm, 24 h after production. However, the maximum optical transparency was 71.8% at 400–800 nm immediately after production. The optical transparency of the hydrogel reached maximum after 24 h of its production because the stability of the structure increased over time via the mobility of the hydrophobic association, and the hydrophobic bond changed from an uneven state to a uniform state [26]. It was confirmed that the transparency of #4 hydrogel after 48 h was higher than that of freshly formed hydrogels.

The hydrogel showed 78.5% transparency after one week and 75.2% after one month at 400–800 nm. The long-term durability of the transparency was owing to the stable chemical structure, as observed from the FT-IR data in Fig. 2a, in which the peaks of the as-prepared and one-month-old samples are the same, thereby reflecting the stability of the chemical structure.

#### Antifreeze Property

Hydrogels are considered promising for wearable devices owing to their flexibility and biocompatibility; however, their high moisture content poses practical challenges related to freezing [32]. Refrigeration results in a loss of wearable properties and electrical conduction. Many studies have aimed to confer antifreeze properties to hydrogels and found that the freezing point decreased when glycerol or ions were added [18, 33]. Herein, glycerol, NaCl ions and SBMA were added to reduce the ice crystallization point. The DSC graph shows no peak values except for the sample without glycerol, NaCl ions, and SBMA, which indicates that the low-temperature resistance of the samples in this study was extremely high (Fig. 3c). Figure 3d shows that the hydrogel has antifreeze properties at − 17 °C when NaCl ions or glycerol or SBMA was included. Furthermore, #4 hydrogel could sustain stretching, twisting, and other flexible movements, confirming that ice was not crystallized. However, the hydrogel without SBMA, glycerol, and NaCl froze at − 17 °C. Figure 3e shows that even when a circuit was configured with a hydrogel stored at − 17 °C for 6 h, the LED lit up. At a strain of 10%, it is confirmed that the sensitivity did not change even when stored for a long time (16 h) at a low temperature (Fig. 3f).

#### Self-healing

**Fig. 4:**
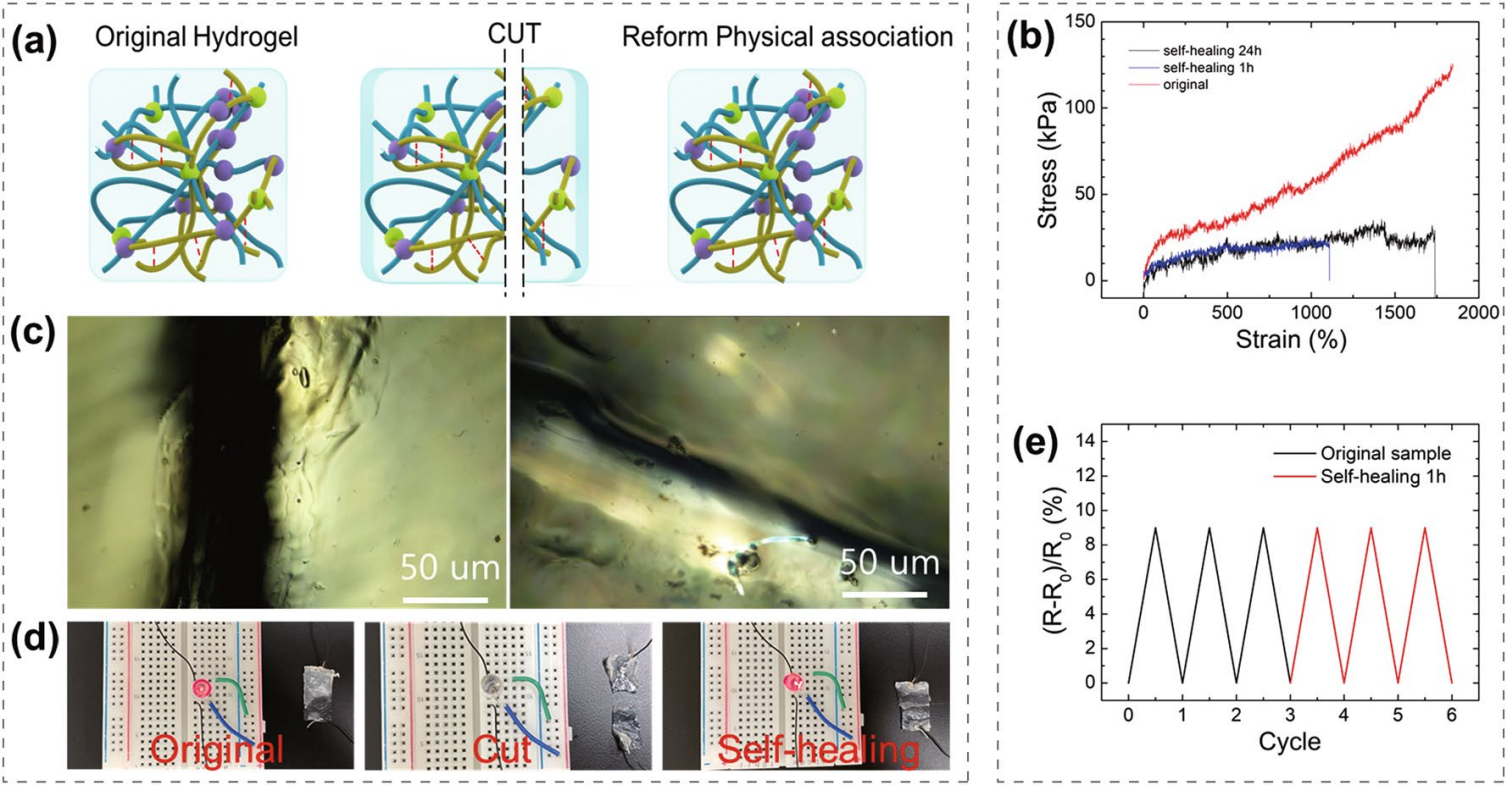
Studies of #4 hydrogel: **a** Mechanism of self-healing. **b** Stress–strain curves of the original and self-healing samples. **c** Microscopic images of the self-healing process. **d** Light change of an LED during the cutting/healing process. **e** Sensitivity graph of the original and self-healing samples

Hydrogels have a self-healing ability, that is, the physical bond is reversible, as shown in Fig. 4a. When cross sections of a cut hydrogel are connected, the micelle forming the physical association reaggregates through its mobility due to the hydrophobicity of micelles in the hydrogel [26]. This self-healing ability can extend the life of the hydrogel by efficiently compensating for the damage to the sensor.

This theory is confirmed by mechanical tests (Fig. 4b). The hydrogel exhibited a strain rate of 1000% 1 h after it was cut. After 24 h, the strain increased to 1738%, indicating that the hydrophobic association is continuously recovering. However, the self-healing hydrogels showed a lower strength than the original sample, which is believed to be caused by the disconnection of the non-reversible chemical association. Furthermore, it is possible to observe the self-healing ability through a microscope (Fig. 4c). The crack width decreased after 24 h. Figure 4d, e show that the electrical properties of the hydrogel after self-healing were the same as those before cutting. As shown in Fig. 4d, the LED turns off after the hydrogel is cut in half. However, it is subsequently relit because the self-healing hydrogel forms reversible association chains where conductive ions can cross the interface. At a strain of 10%, the sensitivity values of the original and self-healing samples were the same (Fig. 4e). In addition, the conductivity was also remained constant, confirming that the electrical performance of the self-healing hydrogel was not affected as a strain sensor (Fig. S5).

### Electrical Properties of the Hydrogel Sensor

**Fig. 5:**
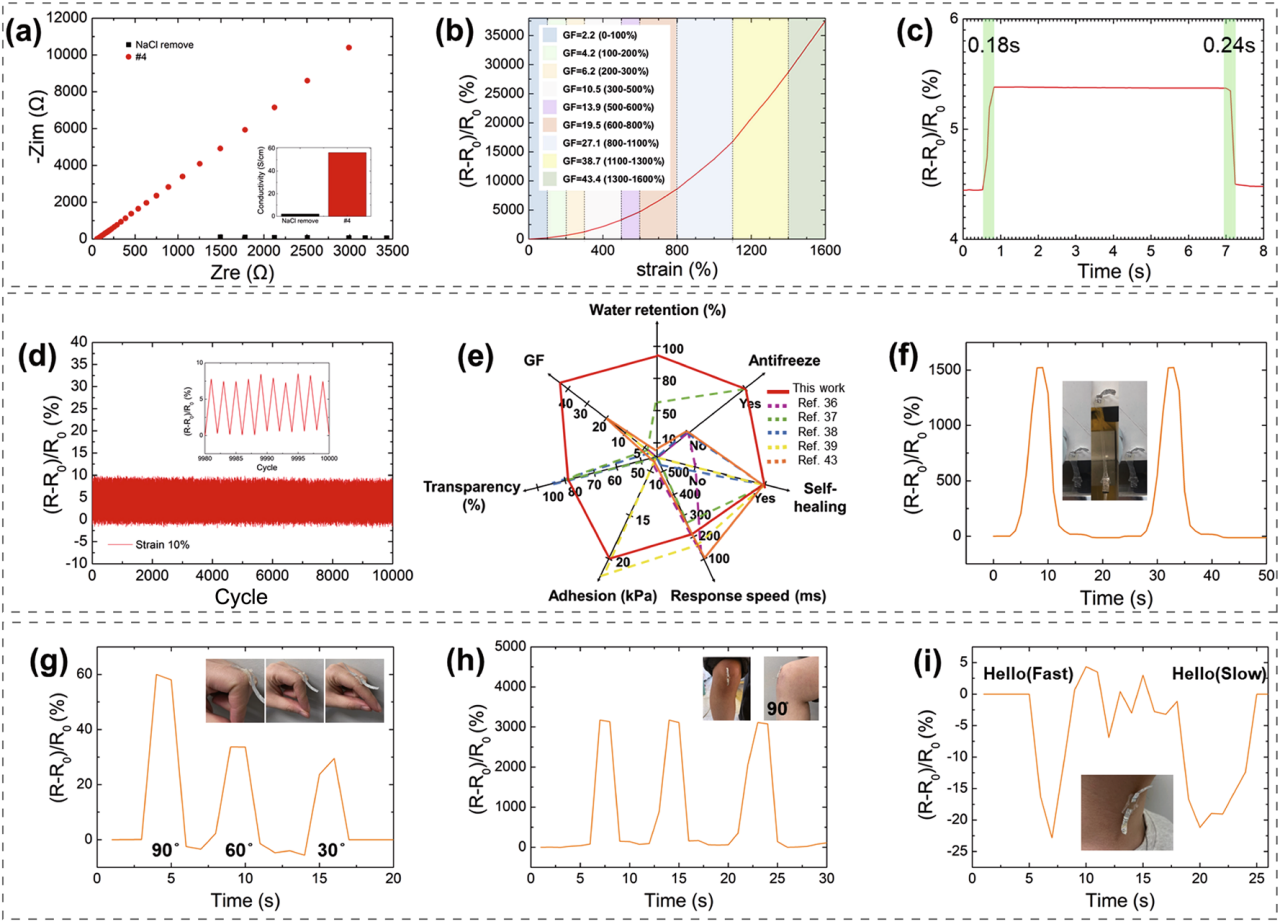
Sensing performance of hydrogel #4: **a** Nyquist plot of hydrogel #4 and hydrogel #4 without NaCl, and conductivity of hydrogel #4 and hydrogel #4 without NaCl. **b** Strain–sensitivity curve and GF. **c** Response curve for stretching and returning. **d** 10,000 cycle tests at a strain of 10% with an enlarged graph showing the last 20 cycles. **e** Heptagonal performance graph comparing the hydrogel #4 to previously reported hydrogels. Applications of the hydrogel sensor for the movement of the **f** window, **g** finger, **h** knee, and **i** vocalization

The multiple-crosslinked P(SBMA-co-AAm) hydrogel has a high conductivity and excellent mechanical strain sensing ability; therefore, it can be used as a strain sensor. In Fig. 5a, the hydrogel #4 diverges through the Nyquist plot, proving that it is an electrically stable material. An unexpected difference in conductivity was observed between the hydrogel without NaCl (2 S cm^−1^) and hydrogel #4 with NaCl (56.2 S cm^−1^). As shown in Fig. 5b, it has a minimum GF of 2.2 at the strain of 0–100% and a maximum GF of 43.4 at the stain of 1300–1600%. The GF reflects the slope of each section, and it increases with the strain percentage because the resistance increases rapidly as the path along which the ions move narrows. The slope (GF) of the graph is also observed to increase as the strain value increases, as 100% of the late strain (ex: 1600–1700%) has a greater effect on the ion migration than the 100% (ex: 0–100%) of the early strain. Almost all hydrogel strain sensors showed the same phenomenon [34, 35]. In addition, the reason why the curve shows a more linear section toward the higher strain may be related to the breakdown of non-covalent bonds in the hydrogel internal network. At lower strain, non-covalent bonds such as hydrophobic associations are broken in large numbers, resulting in varying slope changes; when the strain increases, the number of broken non-covalent bonds decreases, resulting in a more linear curve. The response time of the sensor was 0.18 and 0.24 s for stretching and returning, respectively, which are considered high speeds. Additionally, the difference in reaction speed between stretching and returning was 0.06 s, showing negligible hysteresis (Fig. 5c). Figure 5d presents the cycle stability of the sensor. The durability of the sensor significantly affects its practical applications. The input and output waveforms of the sensor show reproducibility over 10,000 cycles. These results indicate that the durability and reliability of our hydrogel sensor are sufficient for long-term application. Figure 5d shows an enlarged view of 20 cycles of the stress durability curve at a strain of 10%; the curve repeatedly shows similar patterns.

The P(SBMA-co-AAm) hydrogel was compared to those of previous studies in a heptagon graph of GF, water retention, self-healing, antifreeze, transparency, and response speed (Fig. 5e). The P(SBMA-co-AAm) hydrogel demonstrated superior multi-functional abilities than the previous studies, which could only partially achieve these abilities [36–39, 43]. Figure S6 shows that the GF, which is the core function of the strain sensor, of the hydrogel developed herein is much higher compared to the previously reported hydrogels [18, 19, 40–43]. In addition, compared to the hydrogel consisting only of AAm (Fig. S7), addition of SBMA has improved the hydrogel properties, which highlights the importance of SBMA. SBMA provides more polymer networks and ion carriers than AAm due to the electrical bonds between the SBMAs. Therefore, even with the same strain, the internal density of the network is much higher and changes more greatly in the hydrogel with SBMA. It is estimated that the resistance change rate is higher than that of the initial stage due to the reduction of the ion carriers. In fact, it has been reported that the change of the network according to this strain change also affects the conductivity. In addition, it showed a change in sensitivity according to the content of the hydrogel [29]. Therefore, these findings strongly suggest that the as-developed hydrogel can be applied as an ultrasensitive strain sensor.

Because the hydrogel exhibits stretchability and self-adhesion properties without chemical adhesives, it could be attached to human joints to monitor their movements in real-time. Owing to the high adhesion, the gap between the sensing object and the hydrogel sensor can be minimized, and an accurate signal can be received, thereby reducing the noise and increasing the sensitivity. Furthermore, owing to its high tensile strength, it can also be used to capture the movements of doors and windows (Fig. 5f), as well as the movements of furniture, which demonstrates the possibility of its usage in a smart house. Figure 5g shows the attachment of the hydrogel to the joint of the finger to track the movement and the bending angle. As the angle increases, the hydrogel is stretched, and its resistance increases. The same mechanism can also be observed when tracking the knee movement (Fig. 5h). In addition, the hydrogel could also be used to sense sound signals when attached to the neck (Fig. 5i). When saying “hello,” the graph shows the same peak points but different waveforms for fast and slow pronunciation.

## Conclusions

Herein, we produced an ionically conductive multiple-crosslinked P(SBMA-co-AAM) hydrogel through a one-pot synthesis method. This hydrogel exhibited water retention, antifreeze properties, self-healing, and transparency, with improved strength, good adhesiveness, and a high GF. Glycerol and NaCl ions were incorporated to confer moisture stability (e.g., water retention) and antifreeze properties. The synthetic route used chemical bonds to structure the basic backbone of the hydrogel, and the physical bonds formed from the micelle afforded elasticity, self-healing, enhanced strength, and transparency. The transparency enabled visibility, and the material characteristics were maintained for approximately one month. Zwitterionic SBMA was added to the hydrogel to provide it with adhesive ability and increase ion mobility. The resulting hydrogel exhibited high sensitivity, with a GF of 43.3 at a strain of 1300%–1600%, which is more than twice of the sensitivity of the previous reports, considerably enhancing its sensing performance. We have demonstrated that it could detect various human movements and potentially be used as a wearable human body monitoring sensor. It can also be used as a sensor for indoor facilities and furniture, such as capturing the movement of windows owing to its high strain, which indicates the possibility of its usage in smart house applications. The proposed hydrogel overcomes the limitations of previously reported hydrogels and can significantly promote the development of hydrogel devices. The multiple-crosslinked P(SBMA-co-AAM) is promising for applications in wearable electronics, smart house, tissue engineering, and soft robotics. However, after self-healing, the strength of the sensor rapidly decreases. Although the self-healing hydrogel demonstrated a strain of 1000% and constant sensitivity and conductivity, further improvement for the strength will allow the fabrication of more durable sensors. Therefore, follow-up studies are required to address this aspect.

### Supplementary Information

Below is the link to the electronic supplementary material.

Supplementary file1 (PDF 303 kb)
