# Supplementary material for: Ultrasensitive and Highly Stretchable Multiple-Crosslinked Ionic Hydrogel Sensors with Long-Term Stability
Source: Nanomicro Lett. 2023 Feb 15;15:51. doi: 10.1007/s40820-023-01015-7 (PMC9931967; doi:10.1007/s40820-023-01015-7)
Supplement: Supplementary file 1 — Supplementary file1 (PDF 303 kb) [file 40820_2023_1015_MOESM1_ESM.pdf]

Supporting Information for

Ultrasensitive and Highly Stretchable Multiple-Crosslinked Ionic Hydrogel Sensors with Long-Term Stability

Jin-Young Yu[1], Seung Eon Moon[2], Jeong Hun Kim[2, *], and Seong Min Kang[1, *]

[1]Department of Mechanical Engineering, Chungnam National University, Daejeon 34134, Korea

[2]Emerging Nano-Materials Research Section, Electronics and Telecommunications Research Institute, Daejeon 305-700, Republic of Korea

*Corresponding authors. E-mail: smkang@cnu.ac.kr (Seong Min Kang), jeonghun@etri.re.kr (Jeong Hun Kim)

Supplementary Figures and Tables

Fig. S1 Hydrogel composition

Recipe	Monomer	SBMA	AAm
#1	g	0.000	1.200
	m	0.000	5.627
#2	g	0.276	1.129
	m	0.329	5.298
#3	g	0.553	1.059
	m	0.660	4.967
#4	g	0.838	0.986
	m	1.000	4.627
#5	g	1.106	0.918
	m	1.320	4.307

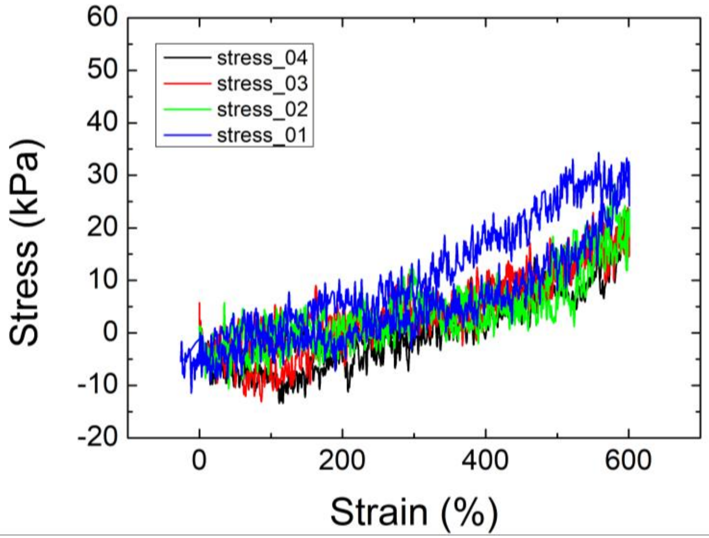

Fig. S2 #4 Stress–strain curve. Reciprocating stress was measured for a strain of up to approximately 600%

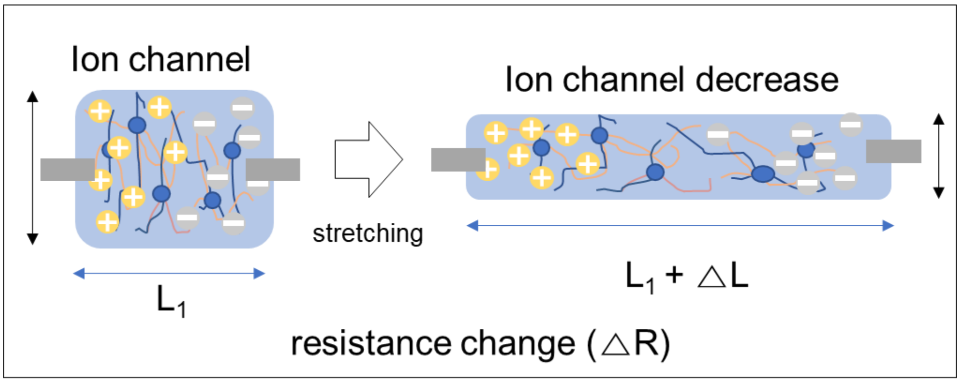

Fig. S3 Principle of hydrogel strain sensor

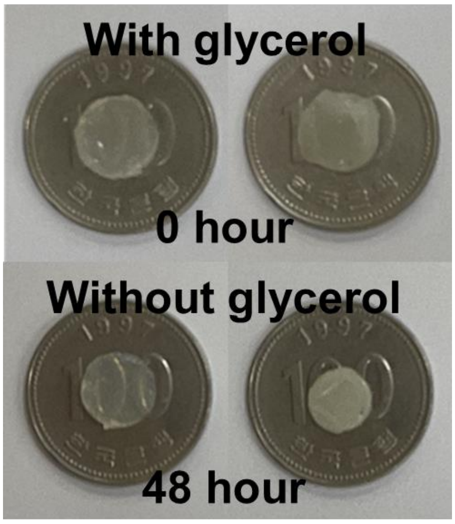

Fig. S4 Volume change of hydrogel #4 and hydrogel #4 with glycerol removed over 48 h

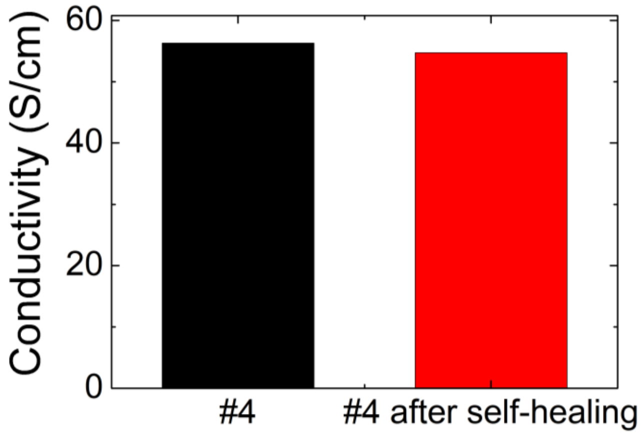

Fig. S5 Conductivity of hydrogel #4 before and after self-healing

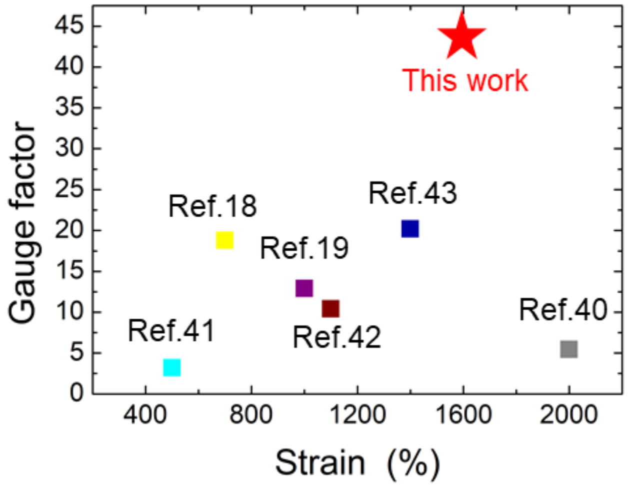

Fig. S6 Comparison of GFs of multiple-crosslinked P(SBMA-co-Aam) hydrogel sensors at different strain with those previously reported sensors

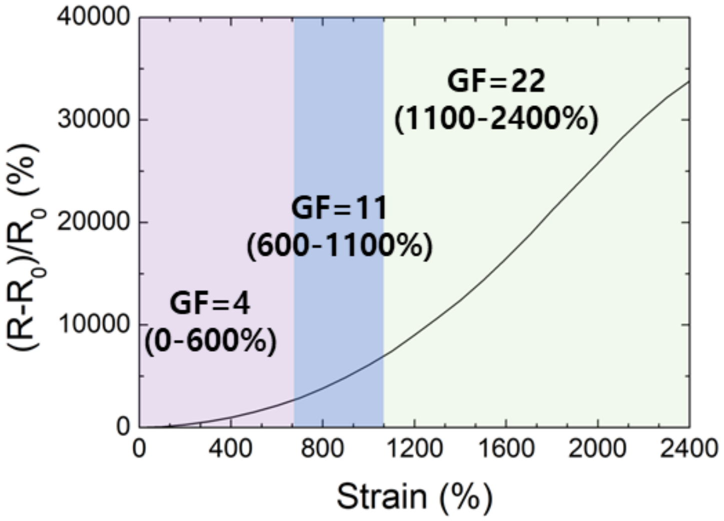

Fig. S7 GF of multiple-crosslinked P(SBMA-co-AAm) #1 hydrogel sensors at different strain sections
